# Jottings

**Published:** 1910-03-12

**Authors:** 


### Jottings.

The King visited King Edward VII.'s Hospital for Officers, in Grosvenor Gardens, on March 1.

The annual meeting of University College Hospital will be held on Thursday, March 17 next, in the board-room at the Hospital, Gower Street, W.C., at 3.30 p.m. precisely.

The annual banquet of the French Hospital will be held at the Hotel Cecil on Saturday, April 30; H. E. the French Ambassador, will preside, supported by the Lord Mayor and Sheriffs of London.

Princess Louise (Duchess of Argyll) has given her patronage to the special *matinée* at His Majesty's Theatre on May 23 in aid of the Royal Waterloo Hospital for Children and Women and the Infants Hospital.

The 32nd ordinary general meeting of the Home Hospitals Association for Paying Patients will be held at Fitzroy House, 16-18 Fitzroy Square, W., on Thursday, March 17, at 4 p.m., Sir Henry Burdett, K.C.B., in the chair.

Presidents and lady presidents are reminded that March 1 was the latest date for sending in recommendations to 29 Southampton Street, Strand, W.C., for grants to country hospitals. Inconvenience has been experienced by the non-receipt in many cases.

Princess Victoria paid a surprise visit to the King Edward VII. ward of the Royal National Orthopædic Hospital on Monday last. She stayed for an hour, and her visit gave the greatest pleasure to the children. The secretary and the matron had the honour of escorting her Royal Highness round the institution.

The increase in the number of cots and beds from sixty to ninety at the Royal Waterloo Hospital for Children and Women has made the need for new subscriptions imperative. At the annual meeting, held at the Mansion House, the Lord Mayor supported a resolution commending the hospital to the peculiar support of the City.

At the annual general meeting of the governors of the Italian Hospital, Queen Square, W.C., it was resolved: "That the cordial thanks of the governors be, and are hereby, tendered to the editors of the British and Foreign Press and Press agencies for their continued courtesy and greatly valued help to the hospital during the past year."

A proposed sanatorium for consumptives under the county authority for Middlesex having come to nothing, a movement has been started to provide such an institution by private enterprise. At the opening meeting the Hon. C. T. Mills, M.P., presided, and a further meeting is to be held at Hounslow shortly, when an address will be given by Sir Douglas Powell, P.R.C.P.

Hospitals in the County of London or within nine miles of Charing Cross desiring to participate in the grants made by King Edward's Hospital Fund for the year 1910 must make application before the 31st inst. to the Hon. Secretaries, 7 Walbrook, London, E.C. Applications will also be considered from convalescent homes and sanatoria for consumption which are situated within the above boundaries, or which, being situated outside, take a large proportion of patients from London.

